# Exploring the Barriers: A Qualitative Study about the Experiences of Mid-SES Roma Navigating the Spanish Healthcare System

**DOI:** 10.3390/ijerph15020377

**Published:** 2018-02-22

**Authors:** Emilia Aiello, Ainhoa Flecha, Olga Serradell

**Affiliations:** 1 Community of Researchers on Excellence for All (CREA), University of Barcelona, Avinguda Diagonal 690, 08034 Barcelona, Spain; emilia.aiello@ub.cat; 2 Department of Sociology, Universitat Autònoma de Barcelona, Campus de la UAB, Edifici B, 08193 Bellaterra, Barcelona, Spain; olga.serradell@uab.cat

**Keywords:** Roma health, healthcare cultural sensitivity, healthcare barriers, Roma women, Roma values

## Abstract

Whereas the topic of the ‘cultural sensitivity’ of healthcare systems has been addressed extensively in the US and the UK, literature on the subject in most European countries, specifically looking at the situation of Roma, is still scarce. Drawing on qualitative research conducted mainly in the city of Barcelona under the communicative approach with Roma subjects who have stable socioeconomic positions and higher cultural capitals (end-users, professionals of the healthcare system, and key informants of a regional policy oriented to the improvement of Roma living conditions), the present study aims to fill this gap. We explore the barriers that the Roma face in accessing the healthcare system, reflecting on how these barriers are accentuated by the existing anti-Roma prejudices and institutional arrangements that do not account for minority cultures. Our results point out a series of obstacles at two levels, in the interaction with healthcare professionals, and in relation to existing institutional arrangements, which prevent Roma families from having equal access to the healthcare system. Education stands up as a mechanism to contest anti-Roma sentiments among healthcare professionals.

## 1. Introduction

Another issue that is not understood is that we have a different notion of what a nuclear family is. If non-Roma are happy saying that your family is your parents and your children, that’s great. But we are not the same. So, if you are a nurse [you need to know], it is of equal importance for me that in the hospital, at the waiting room there are my parents, my children, but also the sister of my parents. And if they are many… imagine, 4 and 4, with their husbands—because they are my uncles—with their children, because maybe I want to say goodbye to them also, because I’m dying! [she explains a hypothetical situation] Well, you might have 20 people there. But people always complain… [referring to some health-care professionals and hospital staff]. Of course, at the organization level I understand… It’s right, I know that there are 2 beds in each room, well I know that maybe having 20 persons next to you can be annoying, and it is also annoying for the Roma person that is dying… But no worries, I would tell my family to be quiet as far as possible…. It’s not a problem, we should be allowed to stay there…! When possible, if we don’t bother anybody, why not? Why we can’t stay there accompanying our relatives? If a non-Roma wants to stay alone, or just with another person, I understand that…. But for me my family are not 4 people, are 20 (…) (Anne, Romani woman).

Anne’s narration illustrates a generalized experience among many Roma families in Spanish hospitals. Most Roma, when they have a relative hospitalized due to a serious illness or risk of death, want to spend as much time as possible with the person and thus accompany the family during these hard times. As Anne explains, for her, family does not mean the nuclear family, but relatives with whom she organizes her life or who she identifies with her home, her culture, or her way of doing things. This manner of care relations reaches beyond the nuclear family towards a more extended one. However, because of how hospitals are arranged in Spain and in many Western countries, situations like Anne’s can often generate tensions or make other patients or health professionals uncomfortable in the management of certain locations (for example, waiting rooms, shared rooms, or hospital halls) or in relation to the family members present (for example, children or elders). It is clear that the system is currently not prepared to accommodate Anne’s family needs and fully realize their right to access to a quality health system. These unresolved questions emerge as fuel for anti-Roma sentiment in a context where formal access is a partial reality because of this lack of institutional adjustment. Even though the health system is supposed to be neutral or color blind, it is de facto far from being so. Thus, the health system increases the perceived distance between the Roma and health institutions, as even though it formally serves this constituency, it does not do so fully.

The present article introduces qualitative research conducted in Barcelona about the invisible barriers that hinder the Roma’s full access to the Spanish healthcare system. It highlights a series of obstacles at two levels, in the interaction with healthcare professionals and in relation to existing institutional arrangements, what prevent Roma families from having equal access to the healthcare system.

While we know much about Roma health inequalities and the role played by the social determinants of health [1,2], specific barriers in the everyday experiences of the Roma with the healthcare system in Spain remain understudied in the academic literature. What is more, in Spain, the strata of the population that suffers from the consequences of these barriers include not only those Roma who are in vulnerable situations, but also the ones who hold better socioeconomic positions. Linked to this, the goal of our study is to analyse the barriers that the Roma people face in accessing the healthcare system, deepening the case of those who are not in the most vulnerable social situations, but of those who have stable socioeconomic positions and higher cultural capitals, which can help to better understand how these barriers are accentuated by the existing anti-Roma prejudices and institutional arrangements that do not account for minority cultures.

### 1.1. Background: the Roma in Spain

There is controversy about the exact number of Roma people who live in Spain, since the official census does not collect ethnic data. According to the Foessa Survey of 2007–2008 and 2008–2009, the estimated number of Roma in Spain varies between 800,000 and 970,000 people [3]. Regarding Roma immigrants, a survey conducted by the Fundación Secretariado Gitano in 2011 estimates that there are approximately 125,329 people, mainly coming from Eastern European countries [4].

Data indicates that, in general, in Spain, the Roma community holds a slightly better socioeconomic situation than in other EU countries. However, this ethnic community continues to be highly discriminated and faces high levels of poverty. Regarding the educational level of Roma people, 8.6% are illiterate (in comparison to 2.2% of the total Spanish population); 50.7% do not have a formal education; 18.8% have completed primary studies; 11.9% have some type of compulsory secondary studies but not completed; and 2.6% have a university degree or have finished any other type of high level education [4]. The Roma population is younger than the general Spanish people, with an average age of 28.1 years in comparison to 41.36 for the Spanish mean. Regarding other indicators related to the labour market, a scarce 38.4% of Roma people occupied are salaried. This indicates the weakness of the job positions of the Roma, linked to social unprotection and the absence of labor rights. Moreover, 91.5% of the Roma who are occupied work in the service sector, often in precarious jobs in which less educational and professional training is required. Discrimination is persistent among the Roma in Spain. The same survey reveals that three out of 10 have felt discriminated in the last year solely for being Roma.

Much of the existing evidence has already outlined the poor health quality of the Roma people in comparison to the non-Roma in general in all EU-countries, mainly due to differences in the social determinants of health and thus the inequalities that the Roma experience in accessing good quality education, labour market, housing, health care, and other public spaces that can make a difference to people’s health [5,6]. Relevant data from the Roma Health Report [5] about the health status of the Roma people in Spain reflects that life expectancy for the Roma community is seven years lower than for the general population. Regarding the access to medical insurance, there is no significant difference among the Roma and non-Roma. The Roma people attend general medical check-ups less frequently than non-Roma population. This is coherent with the data that, in general, the Roma consume more medicines than the general population. Other health services that are not covered by the national health system (e.g., dental healthcare) are less utilised by Roma than non-Roma. This data also reveals that approximately 25% of Roma women aged over 16 have never gone to a gynecologist compared with 18% of the non-Roma population, and are more likely to attend for a specific problem rather than in a preventive way. The same trend is observed for mammography checks: 59% of Roma women recommended for mammography have received one, compared to 72% of non-Roma women [5] (p. 107).

### 1.2. Research on Healthcare Systems’ Cultural Sensitivity

Whereas the topic of the ‘cultural sensitivity’ of healthcare systems has been addressed extensively in the US and the UK, literature on the subject in most European countries and in relation to the Roma is scarce [7,8,9,10]. Research efforts have highlighted different treatments in doctor-patient communication in accordance with the patient’s ethnic background. In this regard, a review by Schouten and Meeuwesen found that doctors behave differently when interacting with patients of minority descent and that these patients are less verbally expressive [11]. These authors list five key predictors of culture-related communication problems: cultural differences in explanatory models of health and illness; differences in cultural values; cultural differences in patients’ preferences for doctor-patient relationships; racist/perceptual biases; and linguistic barriers [11] (p. 21). All of these findings concerning ethnic minorities in general have also been observed in studies focused specifically on the Roma [12,13,14,15,16]. However, differences should also be recognised, especially in the current context of anti-Gypsyism [17], a racism that is also operating in healthcare settings. Some particular issues are worth mentioning in this regard.

Jarcuska and colleagues, in their study of the differences between the Roma and non-Roma regarding perceived barriers in the access to health services, found that the Roma are highly associated with a poorer self-rated health status and that a large part of this can be attributed to the difficulties in access to the health system [18]. Similarly, in a study conducted in the city of Ghent (Brussels), Hanssens and others showed the lack of trust within the Roma population, distinct from other ethnic groups, when interacting with healthcare providers and the healthcare system in general as a main deterrent to guaranteeing full access to health [12]. The authors noted that whereas financial constraints, mobility, or language appeared to be the most relevant barriers for most ethnic minorities, the lack of trust in healthcare professionals appeared to be a stronger explanatory factor for the Roma. This is even more important in those European countries where the Roma have suffered humanitarian abuses, such as forced sterilizations among Romani women, in recent history [19]. In this regard, vast research has been carried out regarding the discrimination of Romani women at the time of accessing maternity care. In the systematic review carried out by Watson and Downe, they identified the existence of barriers, and what is more, they found that even in those cases when they are able to access care, they can experience a treatment that is discriminatory based on four layers: ethnicity, economic status, place of residence, or language [20] (p. 1).

In a more recent study, Javenic and her colleagues conducted a qualitative study in order to investigate how racism against Roma women affected their access to maternal health care in the Balkans [21]. These authors’ framework reflects how racism works at three levels: first, *personally-mediated racism*, which referred to the perceptions and interactions with the health system and were mainly situations of poor quality of care and communication problems; second, *internalised racism*, which was linked to psychological factors, including a lack of self-efficacy (e.g., women’s mistrust in the efficacy of raising a complaint concerning the treatment received), low self-esteem, and fear; and finally, *institutional racism*, which included issues related to the social environment and resources, health system accountability, financial issues, or education.

Looking at the case of Spain, more evidence was provided by a study conducted by the Fundación Secretariado Gitano in 2007 [22]. The study emphasizes how the inclusion of cultural difference in the organisation and functioning of the health system must still be addressed in the country as in other EU member states. Targeting the communicational barrier as one of the main aspects, particular issues are mentioned in order to overcome such barriers: a lack of adaption of the messages and information that are conveyed to the Roma; diverging criteria for the evaluation of healthcare; the continuous existence of mutual prejudices reinforced by historical prejudices; and negative personal experiences [22] (p. 88). Besides this and in relation to intimate partner violence (IPV), a study led by Vives-Casas analyzed Romani women’s access to primary healthcare services in cases of IPV in Spain [23]. These authors identified that efforts need to be made to advance an integrated approach that enriches the primary healthcare answer to IPV and to promote specific actions to overcome barriers in access, such as ‘enhancing health staff knowledge and skill for providing culture and gender sensitive care’ [23] (p. 4). In general, the study’s findings are aligned with the existing evidence of discrimination against Romani women in healthcare systems in Europe, specifically in maternity care [20]. Nonetheless, all these investigations emphasize the central importance of social support from the family to address healthcare accessibility problems [24,25,26], accentuating the active role that women play in leading the improvement of their family and community’s living conditions [27], which is also identified in other social groups when facing difficult situations [28,29,30].

All in all, the lack of cultural sensitivity in healthcare systems is often fed by the intolerance, prejudices, and stereotypes of some healthcare personnel towards all non-white clients, accentuated in the case of the Roma people as a result of long-lasting discrimination [14,20,21,22,23,24,25,26,27,28,29,30,31]. Although some efforts have been dedicated to exploring the barriers that the Roma community face in accessing the healthcare system in Spain [32], more research specifically addressing how these barriers are embodied, and looking at the cases not only of those who are in the most vulnerable social positions, but also of those who have stable socioeconomic positions and higher cultural capitals, can help us to better understand how anti-Roma sentiments underlie society, and specifically, the health-care system. The present study is an attempt to fill this gap.

## 2. Materials and Methods

This study is framed under the IMPACT-EV approach (2014–2017) [33], oriented not only to advance the elaboration of new theoretical knowledge, but also to achieve the social impact of research [34]. Moreover, it draws on existing scientific investigations conducted jointly among non-Roma and Roma researchers that have made a step forward in identifying actions that contribute to the improvement of the living conditions of the Roma in different social areas. Examples of these projects are the FP6 INCLUD-ED project [35] and the FP5 WORKALÓ project [36], in which the authors of the present study have participated.

Accordingly, the present study has been conducted using *the Communicative Methodology of Research* (hereinafter, CM), characterized by incorporating the voices of all the actors into the investigation process, especially of those who have been traditionally excluded from the creation of scientific knowledge. Based upon arguments of validity (and not arguments of power), actors and researchers are capable of generating interpretations of the social phenomenon, thus creating knowledge in a dialogical way [37]. The knowledge created can inform social change and contribute to the creation of *dialogic evidence-based policies* [33].

The CM is based on different theoretical approaches in the Social Sciences. Habermas’ theory of communicative action contributes to overcoming the interpretative hierarchy among researchers and social actors, establishing the need to ground this relation on the arguments provided (‘validity claims’) rather than on the academic position of the speaker (‘power claims’) [38]. Schütz’s phenomenology also highlights the importance of the subject’s interpretation [39], and Mead’s symbolic interactionism stresses the way in which interactions can modify how we interpret our social reality [40]. The CM draws upon these theoretical lenses and focuses the attention on the intersubjectivity among the researcher and the ‘researched subject’, and on the process of the creation of meaning through communicative interactions, in which participants reach agreements about the understanding of a concrete social reality. The researchers are expected to bring into this dialogue all the expertise about the developments accumulated by the scientific community on that reality which is contrasted with the experiences of social agents. By contrast, the ‘researched subjects’ are expected to bring into the dialogue their sensations, experiences, and visions in relation to the topic that is being discussed [41]. It is in this sense that the CM recovers Schütz’ idea of ‘lifeworld’ [39], those knowledge categories acquired by the person and which serve him/her to interpret the world. While using the CM, the researcher dives through intersubjective egalitarian dialogue into the subjects’ previous learning experiences, thus understanding his/her own views and how they influence his/her actions.

The use of CM as a method of analysis also allows vulnerable populations to not be at the centre of inequalities-addressed policies, but key informants and designers of transformation in the process of overcoming inequalities. This is in line with the EU *Common Basic Principles on Roma Inclusion, ‘Roma active participation’*, which states that ‘the effectiveness of policies is enhanced with the involvement of Roma people at every stage of the process’ [42] (p. 57).

The CM, in line with other methodologies such as the Action Research, aims at moving beyond traditional methodologies by involving actors in the research process with the goal of leading meaningful social change. However, as Gómez and colleagues have explained in detail [41], they differ in key aspects of the research process such as their different conception on the idea of the transferability of results, the validity criteria used, the data collection and analysis, and the importance given to the theoretical basis.

### 2.1. Study Design

As already mentioned, the main goal of this study is to analyse the barriers that the Roma people face in accessing the healthcare system, deepening the case of those who are not in the most vulnerable social situations, but of those who have stable socioeconomic positions and higher cultural capitals, considered as mid-socioeconomic status (hereinafter, SES) individuals. This criteria can help to better understand how these barriers are related to ethnicity, and thus accentuated by the existing anti-Roma prejudices and institutional arrangements that do not account for minority cultures.

As commonly accepted, we have defined the socioeconomic status of the study participants based upon educational attainment, occupational status, and economic status (income) [43]. As mentioned above, the participants of this study distinguish from Roma individuals in a vulnerable situation, due to the fact that they have stable socioeconomic positions and higher cultural capitals. Considering their familiar origin, they are on the path of experiencing upward socioeconomic mobility since they are the first generation in their families to have access to university and to the labour market with stable job positions.

The study was conducted between January 2017 and January 2018, mainly in the city of Barcelona. We carried out 17 semi-structured interviews with a communicative orientation with different individuals related to the focus of the research. Study participants were recruited following a purposive sampling strategy. Accordingly, they were grouped following three criteria:

*Group 1. End-users of the healthcare system:* women and men, who provided relevant information about either their personal experience as end-users of the healthcare system, or experiences of their relatives. These interviews were aimed at capturing the barriers that the participants faced when interacting with the healthcare system, considering themselves not just as end-users that could narrate their experiences, but those that could also reflect on them.

*Group 2. Professionals of the healthcare sector of Roma origin*, who informed about the barriers faced by themselves as users of the healthcare system as well as those perceived barriers that other Roma faced, being themselves ‘insiders’ of the system. They provided a double perspective: end-users and insiders, observers from within the system of how Roma users are treated by healthcare professionals. Besides, they provided information about how other healthcare professionals and end-users perceive them when developing their professional task. It should be mentioned at this point that one of the difficulties of the study was identifying individuals of this profile, given the low figures of Roma who occupy these professional positions.

Finally, *Group 3: Key informants*. The difference between Group 1 and Group 3 was that besides providing the same information as the study participants from Group 1, that is, as end-users of the healthcare system, individuals of Group 3 were also involved as members of the Renovation Team (advisory body) of the *Integrated Plan for the Roma in Catalonia* [44], a regional policy implemented by the Department of Work, Social Affairs and Family of the Catalan Government. Since its first edition in 2005, the PIPG develops a set of cross-cutting actions (including the field of health) with the aim of overcoming the inequalities that the Roma people face, while maintaining the goal of promoting and recognizing this group’s cultural traits. These interviewees could reflect on the barriers the Roma face at the time of accessing the healthcare system from a wider perspective, given the role they develop in the PIPG as they are in contact with many Roma across the territory, and they deal and manage with complaints raised by Roma in different areas.

Thanks to the communicative approach used in the study and the relationship built with the Roma community through previous researches, it was not difficult for us to identify participants for the study. This has been one of the gates of access to the participants in the study. Our professional collaboration with institutions that work with the Roma, such as the PIPG, has facilitated another gate of access to the profiles required for the study. Far from what some could argue, this close relation with the Roma community does not position ourselves and the present study in a subjectivist perspective, neither does it affect our objectivity: semi-structured interviews were conducted following the communicative approach, that is, having an egalitarian and intersubjective dialogue with participants, inquiring on the topics on which we wanted to know about—as a two-way conversation, in which both the researcher’s and the *researched’s* initial view would be changed throughout the conversation due the interaction and the arguments brought into it.

The interviewees’ profiles are as follows:

### 2.2. Data Analysis

All semi-structured interviews were transcribed and, along with the other documental data, were analyzed according to a coding scheme derived from the literature review and the research question.

The communicative methodology deepens the analysis of the *transformative* and *exclusionary* dimensions [45]. Drawing from this approach, for analysing the information gathered, we defined the *exclusionary dimensions* as those actions, interactions, procedures, and structural elements within the healthcare system that constitute barriers for the Roma’s experience of full access to the healthcare system while being sensitive towards their cultural values. Accordingly, the *transformative dimension* was defined as those actions, interactions, procedures, and decisions made within the healthcare system in general that contribute to the advancement towards a more culturally sensitive healthcare system. In addition to the exclusionary/transformative axis, the distinction between subjects (referring to interactions in the healthcare system: the treatment by healthcare professionals and other workers of the healthcare system) and systems (institutional arrangements within the healthcare services) was considered.

### 2.3. Bias and Limitations

In the study, we have worked by ensuring to avoid a source of bias which can hamper the validity of our findings. As seen in Table 1. Study participants profile, nine study participants work as either Roma mediators or in non-governmental organizations. Given their close relation with Roma people from different constituencies and also as they would be motivated to protect their environment, these participants would have tended to reaffirm the Roma vulnerability in accessing the healthcare system. If this was the case, it would be a source of bias that needed to be addressed. Bearing this in mind, we decided to classify study participants into three groups (Group 1, Group 2, and Group 3), and additionally, to go deeper in asking specifically about situations that either themselves or some family relatives experienced when accessing the healthcare system, or when dealing with professionals. In the semi-structured interviews, all participants were firstly asked to illustrate specific examples in which they have suffered themselves and then to discuss this in relation to the broader situation of the Roma in Spain. In this sense, as will be observed in the results section, most of the evidences presented here are related to experiences suffered by the subjects who are narrating them.

Two limitations should be acknowledged in our study and related to the generalization of the findings. First, it should be mentioned that all our participants are Roma people who disclose their ethnicity and live according to their cultural values. In this sense, there might be other Roma people of similar socioeconomic characteristics (mid-SES, with academic credentials) who do not experience their ethnicity in the same way, and do not reveal their Roma background. In not self-identifying as Roma, these individuals might not be identified as such and thus might not encounter the barriers analysed in this study. Therefore, findings of this study are valid to the extent of those Roma individuals who have stable socioeconomic positions and hold educational credentials, and who live according to their Roma cultural values.

The second limitation is related to the scope of the findings and the qualitative nature of the study. Our findings can be considered as valid for the settings and groups studied in the research. We did not seek them to be generalizable to all Roma people of similar socioeconomic profiles navigating different healthcare systems, but to open academic pathways from which new literature can emerge, inspiring other researchers to explore similar situations among the Roma in other European contexts.

### 2.4. Ethics

The present study complies with the Universal Declaration of Human Rights adopted by the United Nations Educational, Scientific and Cultural Organization (UNESCO), the Charter of Fundamental Rights of the EU, and resolution P7_TA 0312 of 9 September 2010, of the European Parliament on the Situation of the Roma People and Free Movement in the European Union [46,47].

All the data collected have been anonymized to protect participants’ identity and personal data. Consent forms were provided to all the persons interviewed with detailed information of the study explaining that they have the right to withdraw from it at any time.

## 3. Results

### 3.1. Barriers Faced When Navigating the Healthcare System

Drawing from the analysis of the different cases and situations explained by our interviewees, in this section, we present and discuss the barriers that the Roma users encounter when navigating the healthcare system. We have identified four elements that act as barriers. Two of them are linked to the interactions with the subjects (interactions with healthcare professionals), namely: biased communication between Roma patients and some healthcare professionals, and stereotypes about Romani women. The other two are linked to the structure of the system (how it is managed), namely: the Roma cultural value of the extended family and the lack of accommodation of healthcare institutions to it, and the management of situations when a Roma dies.

#### 3.1.1. Biased Communication between Roma Patients and Some Healthcare Professionals

Most of the interviewees have shared specific situations in which either themselves or a relative have faced disrespectful attitudes from healthcare professionals. According to our interviewees, common situations that Roma users encounter when dealing with healthcare professionals are that physicians often talk to patients either in a technical way using medical vocabulary, or in other cases, they oversimplify the information as if they were talking to children. As they explain, some healthcare professionals frequently assume that if the patient they are treating is a Roma, no further explanations need to be given ‘as they will not be interested in them’; as they are Roma, ‘they will not have any academic credential and may not have any regular job’. This assumption is fuelled by the persistent anti-Roma sentiment. For the cases of our interviewees, these types of power-based interactions deployed by some physicians are contested when the user, or the person accompanying the Roma patient, reply to the doctor, showing that he/she is also able to understand the medical code.

Miranda, the only one in her family with a university degree, explains how, in two different instances when her father and her sister fell chronically ill, the dialogue between the doctors and the family was not egalitarian but hindered by prejudices towards them:

The most negative experiences were with higher-ranking physicians, with surgeons and with the head of the department. Because I remember that my father had had the colon removed, and for some time, he had to carry a little bag (….) this was very uncomfortable for him, and the physician who was the head of the department was the one who had to teach him how to deal with that. (…) She told my father that she wanted that at least one Roma to go out from the hospital knowing how to [correctly] put on the bag… I mean, treating him like a silly person or worse

(Miranda, 26 years old, holds a university degree)

The experience that Anne explains in what follows is also illustrative of this situation:

You see someone that has not resources, and I’ve seen that myself, the health-care personnel raise up the tone, I mean… is a way of expressing… much more technical, a technical way of communicating… And this happened to me when I attend the doctor with my mother. (…) But when I replied to the doctor in the same way [using the same skills], like: What are you saying to me? Then he replied like if we were having a coffee in my home…

(Anne, Romani woman)

The same occurred in the case of Mateo, who explained how, in his family, he always goes with his relatives when they have a health issue. Mateo’s relatives ask him for help any time that they need to attend important medical appointments as they rely on him to understand complex information that ‘they might not be able’ to understand. He emphasizes how doctors assume this predisposition among the Roma:

The situation changes when I’m there… When I insinuate to the doctor, just without mentioning that I have a degree… but when I say a technical term, just when the doctor realizes that I really understand what he is saying, the way he communicates changes totally, how he addresses to me. (…) The last time that I went to the doctor it was with my auntie, she asked me to go with her and with my uncle because my uncle has had an embolism. This was a complex issue to understand; the explanation that the doctor was giving to us. (…) For her it was difficult to understand all what the doctors say because every time that they go to the hospitals, doctors explained something different. I mean, they explained the same but in different terms. (…) It was the way that doctors speak… I think that this is very important: there was a predisposition among them to think: ‘they are Roma so they will not understand

(Mateo, Roma man)

The following situation explained by Victoria reflects how the initial prejudice against Roma changes when the patients disclose their academic credentials or professional status. Victoria, a young nurse who has worked for several years in different hospitals, narrates a time when, with her daughter at the hospital, she knew what her daughter needed, but the nurse did not expect a “gypsy woman” to be a nurse:

Look, I go to an outpatient clinic and I feel observed…. Maybe they are not looking at me, but I do feel that they are. Sometimes doctors just talk like: this gypsy does not know anything…. But I was: Yes, I have seen them… Because with my daughter, she was born with earwax build-up and I told the person in the hospital: My daughter has earwax build-up. And she said: No, she does not. And I said: Yes, she has, I’ve seen them. And she replied: How can you know if she has earwax build-up? Saying that like Come on, you are a gypsy… And I was: Well, because I have an otoscope at home and I’ve seen them, I’m a nurse and I’m sick of taking earwax, and my daughter has them…

(Victoria, Romani women)

As observed, the nurse was surprised when Victoria replied that she has an otoscope at home. At this point in the interaction, the relationship changes because the nurse realizes that she is interacting with a professional in the same category that she is in. Moreover, Victoria explained how many of her colleagues apparently change their anti-Roma attitudes towards the Roma clients when she intervenes to defend her people. Initially, she does not disclose or hide her ethnicity; only after gaining the confidence of her team does she disclose herself:

When my colleagues realize that I’m Roma they change completely… Also with the patients… Because they want to look good with me… At the very beginning I don’t introduce myself saying Hello, I’m Victoria, I’m Roma. If they ask me of course I say that I’m Roma, but if they don’t ask, I don’t say. But I’m proud of it. If I see that they are saying bad things about my culture, I have to reply. And this used to occur a week after I started to work. So, they already know me and know how I work. So, this has occurred in the past

(Victoria, Romani woman)

This example illustrates how the inclusion of Romani people as professionals in the healthcare system can contribute to overcoming stereotypes and fostering the construction of a more egalitarian and efficient system.

According to Manuel, a Romani doctor with more than 15 years of experience, the kind of treatment that the Roma people receive from healthcare professionals does not depend on their professional category or on the fact that the doctor is Roma or not, but on the vocation and professionality of the person. Manuel emphasizes that healthcare professionals need to be committed with their patients.

However, what the data collected for this study shows is that if these barriers can also be found in other sectors of the general population, such as people with no educational credentials, it is much more acute in the case of the Roma. Marta, a young Romani woman who is currently finishing her degree in law and working in an organization of the third-sector coordinating the mediator service, agreed with Manuel’s views, stressing that conversing with Roma healthcare professionals is indeed a great step forward. Nonetheless, the great challenge is to overcome anti-Roma sentiments among non-Roma healthcare professionals, and to show that Roma users can trust them. This is a general view among all the persons interviewed. In Marta’s words:

If the professional treats you as you deserve, you will attend to him/her, we are not so exclusionary! If I have a doctor that is very competent, and she tells me that I have to go for a check-up, well, I will do it because I trust her and because she is good, regardless of whether she is a Roma or not. Maybe Ok, there would be some families for whom the fact that the doctors are Roma will give them more confidence…

(Marta, Romani women)

#### 3.1.2. Assumptions that Discriminate

The majority of Roma female interviewees agree with the idea that when seeing the doctor, grassroots Romani women are not properly informed about the established treatment protocols, for instance, for gynaecology and obstetrics. They explain that some healthcare professionals take for granted that because they will prioritize the cultural value of virginity, Romani women will refuse any type of gynaecological test.

Victoria explains about a situation when she attended the primary care centre with her daughter:

They had to probe it because I said to the doctor: my daughter does not have a virus [she has an infection], can you please give her a urine test? (…) [The doctor replied]: Well, maybe you don’t like what we will tell you… but if you do not want to go home we will have to probe her. Well, what’s the problem? [Victoria answered] And then she said to me: Ah, I do not know …. because you the *gypsy*… with the virginity… But of course, imagine that the doctor says that to someone that does not know this, the mother might take her baby back home with 40 degrees of fever… Because the doctor has not explained to you. They assume that you’re going to say no

(Victoria, Romani women)

Victoria understood the situation because she is a nurse and already had the information. She complained that if this had occurred to another person with poor health literacy skills, the person may have taken the baby home with a very high fever.

The interviewees emphasize how stereotypes, such as the conviction that the Roma community is more sexist or that Romani women are subordinated to men, also reach the area of health. This has repercussions on the attitude and practices of healthcare personnel. It is observed in the experiences that some of the female interviewees shared where the internalization of these stereotypes leads to professionals making women invisible. This is in the sense that, based on their assumptions, professionals tend to believe that they need to ask for the explicit consent of a man (e.g., husband or father) before following some procedures with Romani women. Again, Victoria exemplifies this type of situation:

There is the perception that Roma men are chauvinistic, and it is not true: ‘because Roma don’t do the dishes, they don’t take care of children…’ They have the stereotype of the chauvinist husband… according to them (non-Roma), the wife is subdued to the man. And so you have to be really careful when treating them, ‘let’s see what your husband says’ [she says that this is what non-Roma think] (…) When I was doing internships in labour rooms, once a Roma woman came with her husband. The one responsible of the section that day was a man gynaecologist, the one in charge of attending the birth. Well, the Roma man was so normal, not expecting a midwife to come. But you see, the doctor started to murmur: You see, now this Roma will tell me something for being a man… Like what they think, that we have a chauvinistic culture

(Victoria, Romani woman)

A similar situation was pointed out by Marta. She explained that while her sister was a girl she suffered of colic, and that many times her parents had to go with her to the emergency hospital. Doctors used to say that what the girl had was something related to the ovaries, so what used to happen was that they gave her some sedative but did not offer her parents any further tests to see what the problem was. According to her, this was due to the prejudice of exploring her, because doctors knew that they were a Roma family, suggesting that this could raise a conflict in relation to the girl’s virginity. However, they did not offer the family any type of alternative, neither explained what options were available:

[Talking about the case of her sister] We never had the option to be revised. They never explained to us…Okay, you have to do the revisions like any other person… Anyone who know the cultural traits of the Roma currently knows the importance of virginity, I understand this, but maybe there are alternatives? If you explain to the families, and if you offer other alternatives… For me, you just check her and do whatever you have to do, health is what goes first… But at any case I do think that you as a professional have to offer alternatives, and this is never done.

(Marta, Romani woman)

As Marta explains, the cultural values of the Roma girls and woman need to be respected, and this does not stand in opposition to the medical healthcare attention. Again, it is observed in these cases how prejudices and a biased image of Romani women as not capable of understanding medical instruction and putting their cultural traits above all else impregnates medical mindsets in many cases. Besides, according to the interviewees, the insensitive attitude of some healthcare professionals, linked to the existing prejudices towards Romani women, affects the way in which Romani women perceive the healthcare system, fostering the perception of health professionals as far from them and not a trustworthy resource where they may seek help.

#### 3.1.3. The (Lack of) Accommodation of Hospitals to the Roma Extended Family

For the Roma people, the family is a central pillar and is perceived to not be the nuclear family but the extended family. When there are health-related issues in the family, relatives want to accompany the ill person as evidence of love and a symbol of family cohesion. In fact, our interviewees explain how Roma can go to the hospital even if they do not know the sick person. Friendship with one of the relatives is a strong reason to come as a sign of support and solidarity. However, this is often considered a problem by the healthcare system, which, based on the dominant culture, focuses on the nuclear family. This tradition clashes with how most non-Roma families behave.

Two elements are identified among the narratives of our interviewees in relation to the Roma cultural value of the care of the family relatives, and the personal experiences derived from them when dealing with healthcare professionals and staff at public institutions. First, the emergence of conflicts or situations of tensions among the Roma families and the healthcare staff, under which underlie anti-Roma sentiments and prejudices. Second, as a contestation from the side of the Roma users, their claim that healthcare institutions need to be accommodated according to their cultural values.

In relation to the first element, many examples were mentioned. For instance, Soledad explained how, although she has never experienced any situation of racism when seeing the doctor, which she attributes to the fact that ‘she doesn’t look like a Roma’, she identifies situations in which together with other Roma people, they have been mistreated in a hospital waiting room, just for being recognized as Roma:

Someone had an operation and we all went to visit him. And you could see in the nurse’s eyes: ‘Uf, yes, they are the Roma… You already have them here’… Okay, I understand that this is a hospital… But the way how they ask you to keep silent, or to leave the place it is not always the most appropriate one

(Soledad, Romani woman)

Antonio, a community leader who had recently been admitted to a hospital, explained how in the hospital he found himself negotiating with nurses in order to be allowed to stay in a bigger room, even in the common room, so he could receive and stay with all those people who were coming to visit him. He explains that, at the beginning, nurses were very reluctant and some of them showed certain prejudices against his friends and relatives. However, once they saw that no disturbance was caused, they started being friendlier.

This was not the case of Marta, who in line with what Soledad explained, thinks that at first sight, what occurs is that when healthcare professionals see that Roma people are together in a patients’ room as visitors, the way in which they are approached is not like non-Roma visitors. She shared a personal experience:

Then also the care in the hospital when you are admitted. I found the case, with my nephew admitted, that we were three people in the room, and in the room next to my nephew’s room there were the same people, and the nurse came only to us and told us: You cannot be that many in the room. I’ll have to call security. And I said: Ok, be quiet. You do not have to call anyone. You just tell and we leave. The people who were next to us were so freaked out…! They couldn’t believe it. These little things happen continuously…. And a hospital belongs to everyone. Because this is also paid with my taxes!

(Marta, Romani woman)

The situation shared by Tomás reflects how it is the assumption among healthcare institutions’ staff that in those cases in which there are Roma involved, some type of conflict will occur and that Roma will react with violence. Furthermore, this is the argument used by some healthcare staff to involve the police, even in cases where nothing has occurred:

There was an accident of a Roma family of my neighbourhood so we all went to the hospital. And suddenly, the police arrived. (…) They stood there to avoid anyone else to enter. People was very nervous and this didn’t help, but there was nobody explaining what was going on… It was simply because of their power: you are not allowed to pass. And I understand that is very difficult to deal with this situation, because 80 Roma were there, standing at the door, but it was not necessary to call the police

(Tomas, Romani man)

Tomas stresses how in this type of situation, the norm is not to engage in dialogue but to apply the ‘hardest’ option.

In these situations, there is no dialogue… The organism [referring to the hospital] doesn’t allow it. I think that if someone from the hospital have come and explained… Or maybe to set a room where we can stay… Come on, it is not the fact in itself, it’s the way how they proceed… We all have common sense. So you can explain yourself better or worse, but we all have common sense

(Tomás, Romani man)

The second element identified is reflected by Iñaki’s example, who emphasizes how hospitals are not accommodated at all to Roma cultural values, specifically in relation to the sense of belonging of the Roma families, and how this is not taken into account at the time of managing spaces within healthcare institutions. According to Iñaki, this is not a problem of the Roma, but a problem of the institution, which must make an effort in order to accommodate cultural diversity:

Hospitals are not well-conditionate to allow the patients to receive visits. And the sense of belonging of the Roma families…. If I know that these are the last days of the ill person, what I want is to spend the maximum amount of time with him. Of course! And if in his room you only put two seats, well, or either you add more seats, or if not, I will bring seats from my home, because as you might understand I will not be standing the entire day? And then, if I bring my seat you will say there you have that gypsy that comes here to obstruct the way… NO, is not that. So you accommodate it (…)

(Iñaki, Romani man)

As the interviewees’ narratives reveal, many Roma users see hospital professionals as behaving with a cold attitude and many times without sensitivity or respect for how Roma experience situations of illness and death; this will be discussed in the next section.

#### 3.1.4. When a Roma Person Dies

In parallel to the issue explained in the previous section, our evidence suggests that the way the health system is organized does not accommodate the needs of the Roma during the process of death. For them, not different from other groups, a family member’s death represents a tragic moment when community support and unity should be made evident [48]. However, the way hospitals and funeral homes are organized in many Spanish cities does not allow for the flexibility to accommodate large numbers of family members on a permanent basis. This situation generates a persistent problem that needs to be addressed.

Anne’s experience reflects that there are many prejudices underlying how the Roma experience death, and she emphasizes that physicians and nurses often do not want to stay in the room when they know that a Roma will die, as they foresee that it could be a ‘problematic’ situation. In her own words, she says:

With the Roma families, there is negligence that can occur at the time of entry of a Roma person into the hospital and also after their death. (…) See, we are very scared that some of the following things can occur. First, they can do something with the body of my relative that I do not authorize. Second, that they keep the body in order to do something without me knowing it. And finally, that I cannot have a wake for my relative in the way I wish. So many Roma families are obsessed with the idea that physicians guarantee and swear that once the person dies, the family will be able to bring the body home. And the physician, because he is really scared, says that of course. (…) But for us what counts is the spoken word; we really trust in it. But if you carefully explain that this should be done by the morgue, that you as a hospital cannot do it, and if you sign a form I will not do any clinical autopsy or I will not extract any organ, then I will trust (…)

(Anne, Romani woman)

In Anne’s example, it is again observed how communication is hampered by stereotypes of the Roma: healthcare professionals often do not explain how the system works, as many of them think that if they do not do what the Roma ask them, it will trigger a violent reaction. Victoria, the Romani nurse mentioned in previous sections, explains how this type of situation is experienced from the ‘other’ side, within the system. With some years of professional experience working as a nurse in different hospitals, she explained how in one hospital, when a Roma was hospitalized and doctors expected that the patient would die, they called the police even before the patient died because they expected that the relatives would react negatively.

Similarly to what the Roma do with the sick, our interviewees explained how their families would throw themselves at their deceased relatives and want to be with them until the last moment. This farewell also takes place in a collective way that incorporates the extended family and friends spending the night together with the deceased as a sign of support, respect, and solidarity. Given that funeral homes offer a day service but close at night, the way in which the Roma veil their relatives is at home. This makes it possible to stay all night long with the deceased person, surrounded by relatives and friends who might come from different parts of the country. However, interviewees stress that this is not a solution as sometimes it becomes a problem for the Roma families, as many people come to the house where the person is being veiled, and conflict situations arise with neighbours who complain of the noise or of other situations:

Some people think we are some savages ‘Making a bonfire in the backyard, at 2 am while we veil someone’. But of course… Get to veil a person in a house of 60 m square! There’s no room to hold a vigil inside a home

(Anne, Romani woman)

Carmen, another of our interviewees, explained the situation she experienced when her father died, this time related to the funeral service:

For us, it is very important… We went to the funeral home, but at 7 pm, they said that they were closing …We didn’t want to leave the place, so they said that if we did not leave the place, they would call the police. So, it is too much that they say this to you in a moment like that (…) [they argued] ‘Because you are Roma, you damage everything and you don’t respect’… [Carmen’s family replied] ‘How come you will call the police? My father is here, and there are few hours left to stay with him.’ (…) So in this type of situation, cultural differences are not taken into account

(Carmen, Romani woman)

The way Jacob, another of the interviewees, elaborates his argument, summarizes in a very illustrative manner the very point of the issue approached in this section: he claims that the Roma should be granted the same rights as the non-Roma, and that offering a 24 h funeral service should be a public service:

It is considered that the fact that the funeral services do not open 24 h is a problem solely of the Roma people. Besides this, at hospitals, when many Roma go to visit someone, we are considered a burden. Well, don’t you consider that this could also be seen as a virtue of the Roma, the fact that we stand together with our sick persons? But no, society just see the negative part. And just to bring into the discussion the religious issue: this looks like the liberal state: ‘social affairs are in charge of the church’. Because the biggest solution for a Roma when a relative die is currently provided by the Evangelical Church of Philadelphia, as it opens its door to the families and allow to do the ceremonies. But come on! This is a service that should be provided by the public system! This is a basic social service and the church should not be the responsible one of providing it. (…) Because the large Spanish society do not claim for that right, the public system will not offer it? Listen, we are also part of this society and have also the right to ask for it

(Jacob, Romani man)

All in all, as observed in this section, the fact that the Roma are not granted the conditions to be able to veil their relatives according to their cultural tradition generates problems with other actors and raises even more stereotypes and prejudices against the group.

## 4. Discussion

Most research on the Roma in relation to their poor health quality has emphasized the constraints within highly marginalized contexts. However, the social reality researched in this study differs from these cases, as show in the case of Roma individuals in stable socioeconomic situations, dealing with a healthcare system to which all of them have access. Individuals interviewed are concerned about the difficulties Roma encounter when navigating through the system because of their ethnic background. In turn, this study illuminates how education becomes a key mechanism to contest anti-Roma sentiments in these situations.

The fieldwork conducted outlines the prevalence of barriers for the Roma at the *interpersonal* and *structural* levels when accessing the healthcare system. Although barriers that other non-native ethnic minorities can encounter such as linguistic issues [11] are not a problem for most of the Roma population in Spain, our findings reveal how the blindness of the healthcare system towards Roma cultural values hinders mid-SES Roma from fully accessing the healthcare system. In what follows, we will explain how this takes place, materializing itself in the four barriers identified in the previous section.

First, it has been discussed how communication between Roma users and healthcare professionals is often biased by anti-Roma sentiments such as discrimination and prejudice. When treating a Roma patient, some healthcare professionals tend to assume that they are dealing with someone with low educational levels or who comes from a poor background. In this sense, many professionals make a direct association considering that Roma patients are not able to deal with basic medical instructions or even understand treatment procedures. The fact that all our interviewees have acknowledged this type of situation, either during situations which occurred to themselves or to their relatives, reveals the biased power relation among the healthcare professional and the patient, based on the ethnic background. Besides this, what our study adds to existing scientific literature is evidence that suggests that holding academic credentials can serve to contest racist attitudes and healthcare power-relationships at an individual level, although anti-Roma attitudes that impregnate the system continue operating.

The second barrier observed is related to the existing discriminatory assumptions about Romani women and how they influence women’s access to the healthcare system, even in cases of mid-SES women. If anti-Roma prejudices and stereotypes are deeply rooted towards the Roma in general, as previous literature evidence indicates, they are still more rooted towards Romani women, which are seen as subjected to men and only dedicated to the care of children [49]. Furthermore, looked at from our communicative approach, some works conducted from a culturalist approach [50] portray Romani women’s bodies as highly sexualized and exoticized, a view which often fosters the western perception of the Roma culture as chauvinist and stereotyped instead of focusing on how Romani women are exerting their human agency in many aspects of their lives [51].

In this regard, our evidence reveals the existence of prejudices among some healthcare professionals about the Roma cultural value of virginity, which leads them to believe that for Romani girls and women, accepting this cultural value is opposed to receiving some types of gynaecological treatments.

Vast literature has reported the barriers that Romani women encounter mainly in the field of access to maternal healthcare [20]. Some of these studies have explained in detail the influence of ‘internalised-racism’ that Romani women can have, that is, how Romani women’s low self-esteem, shame, fear, or actually feeling that they are partially responsible of their health status, internalizing discriminatory attitudes, affects the interaction between healthcare professionals and Romani women [21]. In our study, we specifically point at the embedded racism in the system, linked to a specific cultural value—virginity- vs. the delivery of healthcare.

This assumption made by some health professionals raises two conflicts. On the one hand, not explaining to Romani women the impact that certain decisions can have on their health (e.g., doing or not certain tests; doing some checks-ups), and therefore not giving them the free option to decide. On the other hand, this can lead to altering the quality of health assistance provided in order to (under healthcare professionals’ view) avoid conflict with a cultural value, alleging respect for the difference, which involves falling in a relativistic approach. Our findings do not consider women of being the responsible ones for ‘not asking’ for the information when related to specific aspects of gynaecological health issues, but shed light on the existing constraints derived from the system itself. In this sense, we have explained how some of our female interviewees, women with academic credentials, freely asked healthcare professionals about gynaecological information as they have high health literacy skills; however, it can happen that other Romani women with poorer skills do not find themselves with the same confidence in this type of situation. Some parallelisms can be established in this sense with the study conducted by Andreassen et al. [52]. These authors explored Roma women’s nonparticipation in a free national cervical cancer-screening programme in Romania, looking from the perspective of women themselves, healthcare providers, and policymakers. What is of relevance from this investigation in relation to our findings is that, while the authors found that individual barriers did play a role in the low level of screening attendance, the main barrier reported was that the implementation was conceptualized along the ‘technical superiority’ of the programme, that is, that it was implemented without considering the needs and viewpoints of users [52] (p. 53). What is of concern is that existing prejudices related to Romani-women’s sexuality still impregnate the social imaginary of many healthcare professionals, and this becomes a barrier for Romani women of different socioeconomic situations, holding a higher educational level as a way to ameliorate it. More research needs to be carried out to deepen the analysis of the consequences that this barrier has on women’s health quality.

At the structural level, we have also identified two elements that act as barriers and which the public healthcare system is not accommodating: the value of the extended family, and in relation to this, the way in which Roma deal with death. This aspect is often considered as something negative by mainstream society. As well-explained by our interviewees, the idea that Roma people ‘never die alone’ [48], that is, that Roma are always surrounded by relatives and friends in moments of sickness, rather than being considered as something positive, has often lead to the construction of a social imaginary about Roma as ‘backward people’ that come all together ‘in a tribe’.

This study brings into the scientific literature novel insights about these two aspects, which had not yet been explored in-depth. In this sense, not accommodating these cultural values not only prevents the Roma people from fully accessing the healthcare system, but also contributes to the persisting anti-Roma sentiments, as instead of being considered something that concerns the entire society, it is relegated as a specific Roma issue.

## 5. Conclusions

The present study has shown how the Roma community remains a target of interpersonal and structural racism in Spain in the healthcare system, specifically considering the experiences of some Roma citizens who have been involved in this system as end-users or professionals. Although previous research has already evidenced the existence of stereotypes such as greater predisposition to violence, sexist attitudes, or a lack of interest in education, qualitative research has scarcely explored how these stereotypes underlie the healthcare system in Spain, becoming a barrier in the access of the Roma, including those who are in more stable socioeconomic positions. This study illustrates how some of these widespread stereotypes about the Roma people have an impact on their access to the healthcare system and the quality of care received, and how education can be an essential mechanism to challenge these stereotypes.

Some actions are being implemented in Spain in order to reverse this situation, although they are only having a timid impact at a structural level. An example of this is some of the actions in the field of health that are being developed under the framework of the *Integrated Plan for the Roma in Catalonia* [44], and which will be of major relevance to be analysed in future works. 

A higher presence of Roma people in the healthcare system, as doctors, nurses, or other healthcare personnel, will challenge anti-Roma sentiments by showing that they are also present in these professional positions of prestige, interacting as professionals with their patients and using their cultural skills to generate bonds of trust with Roma clients. All in all, a healthcare system constructed in dialogue with its users is a living system open to accommodating the cultural differences and complexities of the 21st century; a system that recognizes the health needs of its Roma people and takes action to improve its response and therefore guarantees a fundamental right that has been neglected for years.

## Figures and Tables

**Table 1:** Study participants’ profile.

Groups	Profile
Group 1	**Miranda:** 26 years old. Single. Holds a university degree and a Ph.D. Post-doctoral researcher employed in a university.
	**Saray:** 26 years old. Single. Holds a middle-level training diploma in administration and an upper-level training diploma in social integration. Working in a third-sector organisation.
	**Joaquín:** 27 years old. Engaged. Completed obligatory high school. Working in a service company in the public sector.
	**Soledad:** 26 years old. Single. Holds a university degree in Biology and a Master’s degree. Currently following another post-graduate degree. Working as a support teacher in a private academy.
	**Jacob:** 25 years old. Married and father of three children. Working in a third-sector organisation. Currently studying for a university degree.
	**Iñaki:** 33 years old. Married and father of four children. Working as a sociocultural mediator in a local city council. Currently studying for a university degree part-time.
	**Mateo:** 27 years old. Employed in a University Science Lab developing his Ph.D. and developing basic research.
	**Samara:** 49 years old. Divorced and mother of one child. Basic education. Has been involved in the Spanish Women’s Rights Movement for more than 15 years. Currently employed for a city council as a Roma mediator in a public hospital.
	**Toni.** 60 years old. Married, father and grand-father. Has been involved in the Spanish Roma Rights’ Movement for more than 30 years. Holds a university degree. Working as a Roma mediator in a local city council.
	**Raquel.** 30 years old. Single. Studying for a university degree (currently in her last year). Working in a third-sector organization part-time.
	**Tomas:** 39 years old. Married. Currently studying for a degree in the university. Working in Public Administration part-time.
Group 2	**Victoria:** 29 years old. Married. Mother of one daughter. Nurse and also holds a Master’s degree in Surgery.
	**Anne:** 39 years old. Married and no children. Holds a university degree. Has worked as a doctor’s assistant and currently working in a service company.
	**Manuel:** 40 years old. Father of two children. Primary Care Physician. Has worked in different hospitals in Catalonia, one of them located in a mostly Roma-inhabited neighbourhood.
Group 3	**Jose:** 32 years old. Single. Holds a university degree and a Ph.D. Working part-time as a researcher and in public administration in the PIPG. Member of the Renovation Team of the III and IV PIPG.
	**Bettiana:** 38 years old. Mother of two children. Holds a university degree. Social worker employed in a City Council. Is a member of the Renovation Team of the III and IV PIPG.
	**Carmen:** 41 years old. Married. Mother of one child. Currently in the first year of her university degree. Works as a teacher’s assistant in an educational center with a large number of Roma people. Member of the Renovation Team of the III and IV PIPG.
